# Clinical characteristics and long-term outcomes of 101 patients with urea cycle disorders in China

**DOI:** 10.1186/s13023-025-03985-w

**Published:** 2025-08-13

**Authors:** Ziyan Cen, Pingping Ge, Yuhe Chen, Ting Zhang, Peiyao Wang, Lingwei Hu, Benqing Wu, Xinwen Huang

**Affiliations:** 1 Department of Genetics and Metabolism, Children’s Hospital of Zhejiang University School of Medicine, National Clinical Research Center for Child Health, No. 3333 Binsheng Road, Binjiang District, Hangzhou, 310053 Zhejiang China; 2 Children’s Medical Center, University of the Chinese Academy of Sciences-Shenzhen Hospital, Shenzhen, 518106 Guangdong China

**Keywords:** Genetic mutations, Hyperammonemia, Long-term outcomes, Newborn screening, Urea cycle disorders

## Abstract

**Background:**

Urea cycle disorders (UCDs) are a group of rare genetic metabolic disorders characterized by hyperammonemia, which can lead to neurological damage, systemic complications, and even death. Understanding UCDs’ clinical features and progression in the Chinese population will fill research gaps and benefit patients globally.

**Methods:**

This retrospective study evaluated the clinical, biochemical, genetic characteristics, and long-term outcomes in 101 Chinese patients with six subtypes of UCDs between 2007 and 2024. Data were collected from medical records and analyzed.

**Results:**

The overall survival rate was 93.0% among UCD patients. An equal gender ratio was observed in ornithine transcarbamylase deficiency. Newborn screening (NBS) was conducted in this cohort, and 57.0% of patients were diagnosed through NBS. Neurological and gastrointestinal symptoms were the most common. Symptoms often appeared within the first year, especially in the first month. Arginine was the most frequently used treatment, with glycerol phenylbutyrate often used as a nitrogen scavenger in severe cases. Biochemical analysis showed subtype-specific differences, including notable declines in leucine and glycine on low-protein diets. Genetic analysis revealed a wide distribution of mutations, with few hotspots and 17 newly identified mutations. Clinically diagnosed patients had worse outcomes than those diagnosed via newborn screening.

**Conclusion:**

This study is the first to describe the clinical features and long-term outcomes of UCDs in a large sample of Chinese patients, highlighting the importance of newborn screening for early diagnosis and improved treatment outcomes.

**Supplementary Information:**

The online version contains supplementary material available at 10.1186/s13023-025-03985-w.

## Introduction

Urea Cycle Disorders (UCDs), with the estimated incidence of 0.08 to 1.48 per 100,000 live births in China, are a group of rare genetic metabolic diseases, patients often suffer severe neurological damage and other systemic complications, even death due to sustained hyperammonemia [1, 2]. There are several subtypes of UCDs currently known, and N-acetylglutamate synthase deficiency (NAGSD), ornithine transcarbamylase deficiency (OTCD) and carbamoylphosphate synthetase 1 deficiency (CPS1D) are categorized as proximal UCDs. Argininosuccinate synthetase deficiency (ASSD), argininosuccinate lyase deficiency (ASLD) and arginase 1 deficiency (ARG1D) are distal UCDs, while hyperornithinemia-hyperammonemia-homocitrullinuria syndrome (HHHS) and citrin deficiency (Citrin D) are found by deficiencies in two mitochondria transport proteins [3].

The clinical symptoms of UCDs are nonspecific, and the manifestations are hyperammonemia encephalopathy [4]. Early-onset UCDs tend to be the most severe type, which manifest in the neonatal period (≤ 28 days) and accompanied by acute hyperammonemic crises, characterized by poor feeding, vomiting, lethargy, rapid progression to coma, and multiorgan failure, even death, or have severe neurological sequelae in survivors [5, 6]. While the late-onset UCDs exhibit subtler symptoms, which may occur at any age after the neonatal period and are characterized by episodic hyperammonemia, growth retardation, developmental delays, and behavioral disorders [7, 8]. UCDs should be suspected when unexplained encephalopathy occurs in clinical practice, especially in neonates.

Although NBS is mainly used to screen for distal UCDs, the overall survival of UCDs is obviously improved with the implementation of newborn screening and the availability treatments to prevent and reduce the severity of hyperammonaemic episodes [9]. Recommended treatments contains low-protein diet, supplementation with arginine and/or citrulline, and nitrogen scavengers, such as sodium benzoate (NaBz), glycerol phenylbutyrate (GPB) and sodium phenylbutyrate (NaPBA) could effectively improve the prognosis of UCDs [10]. Liver transplantation (LT), often reserved for severe cases, is considered a potentially curative option, particularly proximal defects like OTCD and CPS1D, as it corrects the underlying enzymatic deficiency in the liver [4]. Post-transplant outcomes are generally favorable, with studies reporting 5-year survival rates approaching 90% and rapid normalization of ammonia levels within 24–48 h post-surgery [11, 12], though neurological outcomes remain dependent on pre-transplant damage extent [13]. Living-related liver transplantation has shown comparable outcomes to deceased donor transplantation while offering advantages such as shorter waiting times and better elective timing [14, 15].

Recent studies across different populations have provided valuable insights into the clinical diagnosis and treatment of UCDs. The Japan nationwide study demonstrated that initial blood ammonia level ≥ 360.0 μmol/L was a predictor for poor neurological outcomes, and a significant improvement in the 20-year survival rate under the long-term managements for UCD patients [7]. In Spain, a multicenter study involved 104 UCD cases found neurological sequelae in 52.5% of patients, further underscoring the importance of ammonia control [16]. ASSD was found the most common subtype in India, accounting for 49.6% of cases, with a neonatal mortality rate as high as 63.0% and an overall mortality rate of 70 out of 110 cases [17]. These studies highlighted the critical need for systematic research on UCDs to facilitate in generating early diagnosis and personalized treatment approaches. NBS has increasingly played a key role in early identification and improved outcomes of UCD patients, particularly in regions with widespread implementation. This study, by characterizing the clinical features, treatment, and outcomes of UCD patients in China, will provide an important piece of the puzzle in optimizing global UCD management.

## Methods

### Study participants

**Fig. 1:**
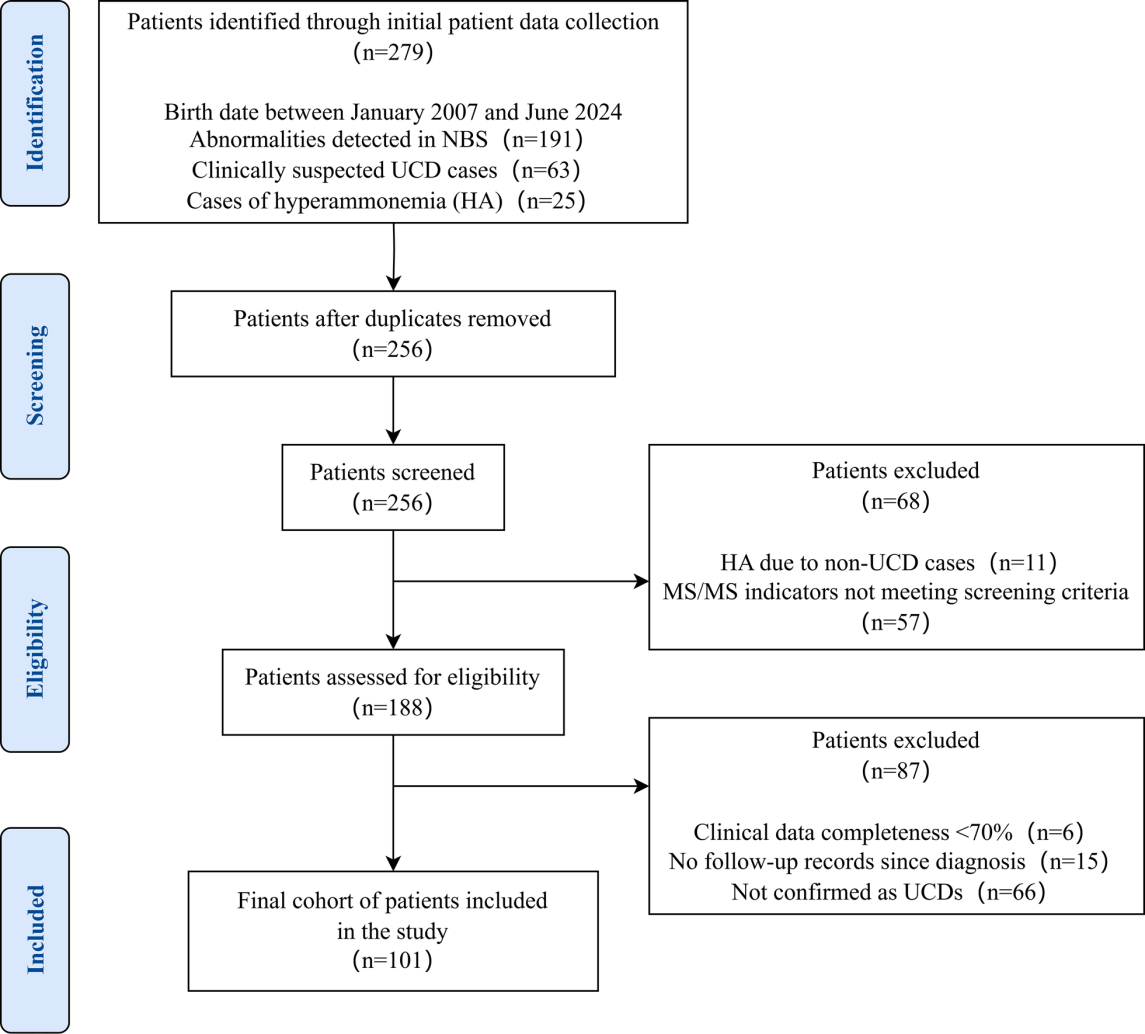
Flowchart of patient selection

This retrospective, single-center study included patients diagnosed with UCDs (excluding Citrin Deficiency) between January 2007 and June 2024 at the Children’s Hospital of Zhejiang University School of Medicine, Zhejiang, China. Inclusion criteria: (1) Patients born between January 2007 and June 2024; (2) Diagnosis as UCDs excluding Citrin D; (3) Have more than one follow-up record after confirmed diagnosis; (4) Have at least 70.0% completeness of clinical data. NBS for selected UCDs has been available at our center since 2008, and has gradually expanded in coverage over time. 279 patients were collected from NBS, suspected UCDs in clinical attendance, and hyperammonemia (HA) onset visited in emergence. After removing duplicate records, 256 patients were screened. Ultimately 101 unrelated patients were diagnosed with UCDs and met the inclusion criteria, were enrolled in the study (Fig. 1). This study was approved by the ethics committee of the Children’s Hospital of Zhejiang University School of Medicine, Zhejiang, China (approval number: 2021-IRB-292). Each patient and/or their parents signed the informed consents.

### Study data and outcomes

Data were gathered across several domains: demographic profiles, clinical features, therapeutic interventions, biochemical measurements, genetic evaluations, and clinical outcomes. A detailed overview of the variables included within each domain is provided in Table 1. The initial screening (pre-treatment) included the baseline data before treatment initiation, while follow-up data were collected post-treatment during the monitoring phase. Reference ranges for protein intake targets and common medication classes (e.g., nitrogen scavengers, amino acid supplementation) are detailed in Table 1. These ranges reflect current standard practice but were individually adjusted in clinical management based on each patient’s tolerance and metabolic control. Temporary enteral feeding (e.g., nasogastric or duodenal tube feeding) was occasionally used during acute episodes to ensure adequate energy intake in patients with feeding difficulties or impaired swallowing. No patients required long-term gastrostomy. Through this data, we would analyze the demographic characteristics of patients, compare clinical presentations, biochemical features, and genetic mutations across subtypes, and explore the disease characteristics of UCDs and their impact on clinical outcomes.

**Table 1:** Overview of data domain and variables collected

Data domain	Variables collected
Demographic profiles	(i) Geographic location
	(ii) Sex
	(iii) Age at diagnosis
Clinical features	(i) UCD subtypes
	(ii) Diagnostic mode
	(iii) Clinical symptoms at presentation
Therapeutic interventions	(i) Low-protein diet (> 1.5 years: 0.8–0.9 g/kg; < 1.5 years: 1–1.8 g/kg)
	(ii) Medication usage
	(a) Citrulline/arginine supplementation (100–200 mg/kg/d)
	(b) Nitrogen scavengers
	NaBz (100–250 mg/kg/d)
	NaPBA (< 20 kg: 100–250 mg/kg/d; > 20 kg: 2–5.5 g/m^2^/d)
	GPB (4.5–11.5 ml/m^2^/d)
	(iii) Liver transplantation status
Biochemical measurements	(i) Plasma ammonia levels
	(ii) Branched-chain amino acids
	(iii) Plasma amino acids
	(iv) Liver function indicators such as AST and ALT
	(v) Hemoglobin levels
Genetic evaluations	(i) Genetic testing results
	(ii) Novel variants (not reported)
	(iii) Family history of genetic conditions
Clinical outcomes	(i) Hyperammonemic episodes (≥ 80.0 μmol/L)
	(ii) Hospitalizations due to hyperammonemia
	(iii) Treatment-related adverse events
	(iv) Long-term outcomes such as growth

### Statistical methods

Statistical analyses were performed on the SPSS software (Version 22.0, IBM Corp., Armonk, NY, USA). Categorical variables were evaluated using the chi-square test, while continuous variables were assessed using either the t-test or the Mann–Whitney U test. A logistic regression model was used to identify independent predictors of clinical outcomes, including survival, liver transplantation, and the use of ammonia scavengers. Z-scores for height and weight were calculated to assess physical growth across age groups. Maps were generated with ArcGIS 10.8.1, and charts were created using GraphPad Prism (Version 10.1.2, GraphPad Software, San Diego, CA, USA). *P* values of < 0.05 were regarded as statistically significant. Bioinformatics analysis used tools like SIFT, PROVEAN, PolyPhen-2, and Mutation Taster to predict the pathogenicity of mutations, with “deleterious” or “pathogenic” variants considered potentially harmful [18–20].

## Results

### Population characteristics

**Fig. 2:**
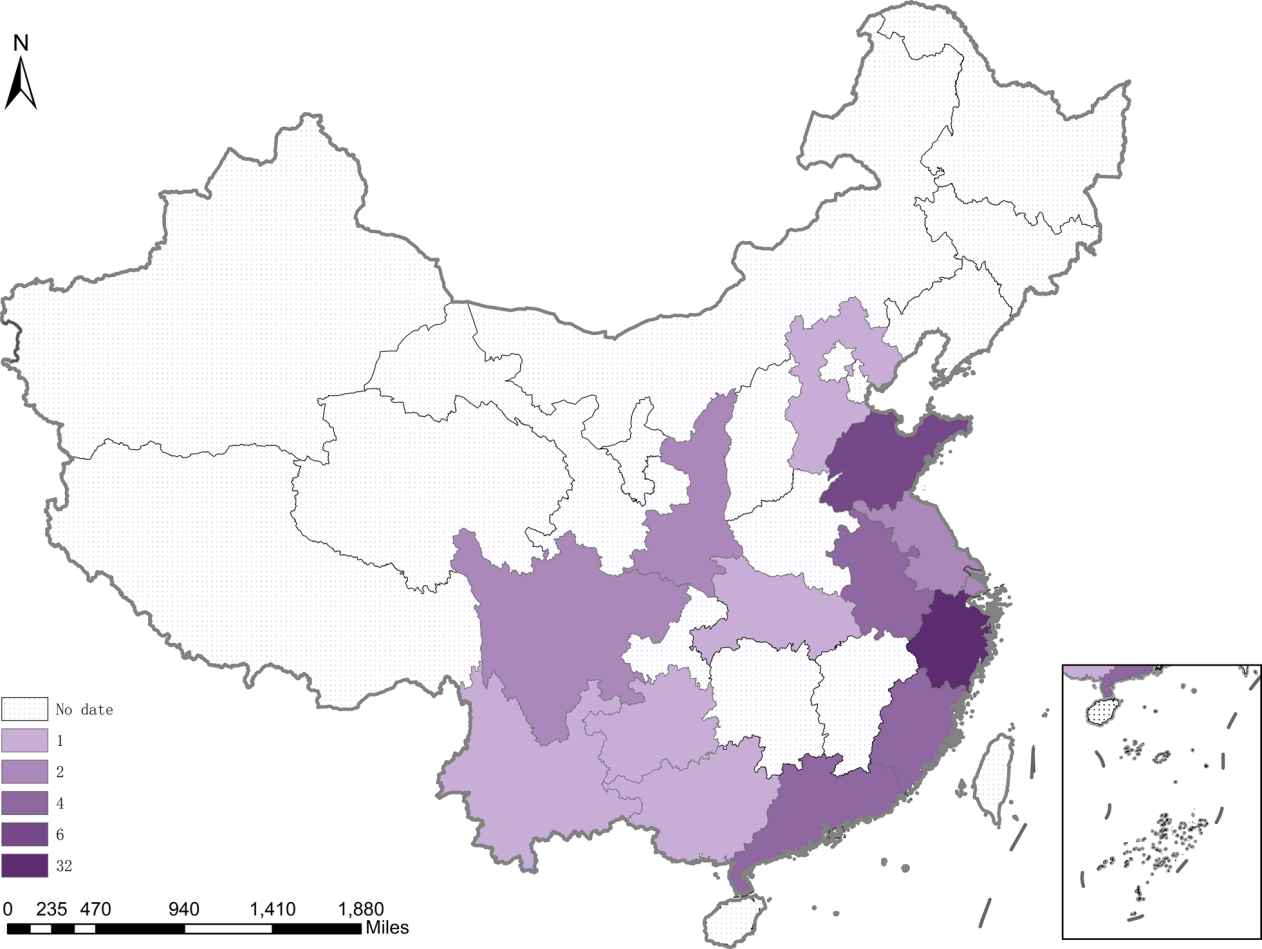
Geographic distribution of UCD cases in China (n = 63). Varying shading intensities represent the total number of cases in each province

Of the 101 UCD patients, geographic data were available for 63 cases, as shown in Fig. 2. Most UCD patients were concentrated in eastern and southern China, with 50.8% (32/63) in Zhejiang province, while northern and western regions reported significantly fewer or no cases (P < 0.001).

The demographic and clinical characteristics of 101 patients (58 males and 43 females) were summarized in Table 2. No cases of NAGSD were identified, 34.0% (35/101) of cases were OTCD being the most common subtype, with male patients with OTCD to female OTCD ratio was almost 1:1, and followed by ASSD (n = 25), CPS1D (n = 13), ARG1D (n = 12), ASLD (n = 10), and HHHS (n = 6).

**Table 2:** Clinical and demographic data of UCD patients

	Patients (n = 101)
Sex, n (%)	
Male	58 (57.0%)
Female	43 (43.0%)
Subtype, n (%)	
OTCD	35 (34.0%)
OTCD Male	18
OTCD Female	17
ASSD	25 (25.0%)
CPS1D	13 (13.0%)
ARG1D	12 (12.0%)
ASLD	10 (10.0%)
HHHS	6 (6.0%)
Mode of diagnosis, n (%)	
Prenatal onset	3 (3.0%)
Neonatal onset	58 (57.0%)
Late onset	28 (28.0%)
Onset before NBS	7 (7.0%)
Family History Onset	1 (1.0%)
Unknown	4 (4.0%)
Age at diagnosis, n (%)	
≤ 1 months	30 (29.0%)
1 months to 1 year	25 (25.0%)
1 year to 2 years	9 (9.0%)
> 2 years	18 (18.0%)
Unknown	19 (19.0%)
Initial symptoms, n (%)	
Digestive symptoms	13 (13.0%)
Neuro symptoms	9 (9.0%)
Lab abnormalities (excluding NBS results)	15 (15.0%)
Unknown	64 (64.0%)
Follow-up duration, n (%)	
< 1 year	27 (27.0%)
1 to 2 years	14 (14.0%)
2 to 5 years	18 (17.0%)
> 5 years	14 (14.0%)
Unknown	28 (28.0%)
Drug therapy, n (%)	
Dietary protein restriction	12 (12.0%)
Citrulline	5 (5.0%)
Arginine	13 (130%)
Citrulline with Arginine	13 (13.0%)
Nitrogen scavengers	24 (24.0%)
NaBz	5
NaPBA	6
GPB	13
Unknown	34 (33.0%)

Of the total cases with UCD, 57.0% (58/101) of cases were diagnosed through NBS (NO group). Another 35 cases, including 28 diagnosed through clinical symptoms and 7 with onset before NBS, were classified into the LO group. Only one case was identified as OTCD through family history when he was 2 years old, he was found directly after his older brother was diagnosed with OTCD. Among the 30 patients diagnosed within 1 month of birth, ASSD was the most common subtype (11/30, 36.7%). In contrast, among the 18 patients diagnosed after 2 years of age, OTCD was predominant (13/18, 72.2%), including 3 males. Supplementary Table 1 presents a detailed breakdown of the age at diagnosis and follow-up duration across all UCD subtypes. The median age at diagnosis was 60 days (interquartile range [IQR]: 30–650 days), with the LO group having a median age of 713 days (IQR 425–1365 days) and the NO group having a median age of 30 days (IQR 26–60 days) (Table 3). Initial clinical symptoms of 37 patients were summarized, 15.0% (15/101) of patients presented with Lab abnormalities, excluding NBS results, such as elevated ammonia or transferase enzymes levels. Gastrointestinal symptoms such as vomiting and diarrhea occurred in 13 (13.0%) patients, and 9.0% of patients suffered neurological symptoms like seizures, including both NBS and non-NBS cases. The median follow-up duration was 2.8 years (IQR 1.1–6.8 years) among 73 patients. Of these, 27.0% (27/101) had a follow-up time of less than 1 year, 14.0% (14/101) of patients were 1 to 2 years, 17.0% (18/101) of patients were 2 to 5 years, and 14.0% (14/101) of patients were more than 5 years (Supplementary Table 1). Most OTCD patients had a follow-up duration of 2 to 5 years, while patients with all subtypes except HHHS included cases with a follow-up time exceeding 5 years. Additionally, all subtypes had patients with a follow-up duration of less than 1 year, with OTCD being the most common subtype in this category (12/27).

**Table 3:** NO vs. LO group comparison of clinical and outcome variables

Variable	LO group (n = 35)	NO group (n = 58)	*P*-value
Age at diagnosis (days)	713 (IQR 425–1365)	30 (IQR 26–60)	*P* < 0.001
Baseline Biochemical Data			
Amon (μmol/L)	129.0 μmol/L	49.0 μmol/L	*P* = 0.008
ALT (U/L)	67.0 U/L	29.5 U/L	*P* = 0.016
Gly (μmol/L)	346.4 μmol/L	454.8 μmol/L	*P* = 0.014
Leu (μmol/L)	106.9 μmol/L	155.0 μmol/L	*P* = 0.001
Use of nitrogen scavengers, n (%)	13/23, 56.0%	8/37, 22.0%	*P* = 0.006
Survival rates, n (%)	30/35, 86.0%	57/58, 98.0%	*P* = 0.032
Liver transplantation, n (%)	2/35, 5.7%	4/58, 6.8%	*P* = 0.936
Neurodevelopmental delay, n (%)	11/20, 55.0%	8/44, 18.0%	*P* = 0.003
MRI abnormalities, n (%)	16/31, 51.6%	11/58, 19.0%	*P* = 0.001
Growth retardation, n (%)	22/25, 88.0%	22/30, 73.0%	*P* = 0.176

Only biochemical variables with statistically significant differences between groups are included to enhance clarity. Median values are presented; corresponding IQR are detailed in the main text due to space limitations. For some variables, denominators are smaller than the total group size due to missing or unavailable data (e.g., 13/23 indicates 23 patients with known data). *P*-value < 0.05 was considered statistically significant

After being diagnosed, treatment data were available for 67 out of 101 patients, with a median follow-up duration of 3.7 years (IQR 1.3–5.7 years). Among them, 13 patients (13/67, 19.0%) had continuous follow-up for more than 5 years, while only one patient had a follow-up duration of less than one month. 18.0% (12/67) of patients treated with low-protein diet alone. Some patients received low-protein diet combined with amino acid supplements, 8.0% (5/67) of patients used citrulline, 19.0% (13/67) used arginine, and another 19.0% (13/67) using a combination of both. In addition, nitrogen scavengers (24/67, 36.0%) was the commonest treatment drug, and GPB (13/24, 54.0%) became the most frequently used drugs in our study.

### Biochemical results at diagnosis

**Fig. 3:**
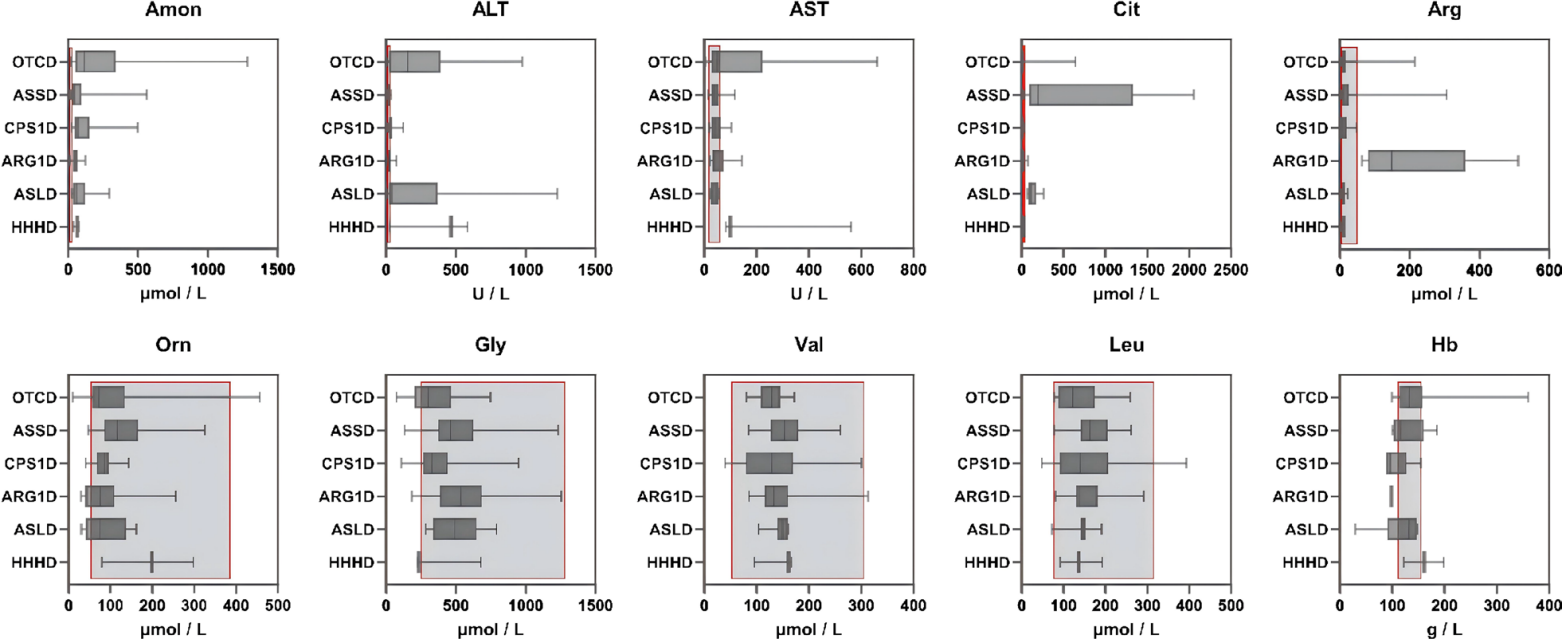
Biochemical results for different UCD subtypes patients. Reference ranges: Amon 9.0–30.0 (μmol/L), ALT 7.0–30.0 (U/L), AST 15.0–60.0 (U/L), Cit 7.14–37.0 (μmol/L), Arg 2.54–50.0 (μmol/L), Orn 52.1–386.6 (μmol/L), Gly 246.6–1283.0 (μmol/L), Val 51.7–305.0 (μmol/L), Leu 75.7–316.0 (μmol/L), Hb 110.0–155.0 (g/L)

Figure 3 presented the biochemical results at time of diagnosis in UCD patients. For a detailed summary of biochemical parameters by subtypes, including median values, IQRs, and patient numbers, please refer to Table 4. The median blood ammonia (Amon) levels was 60.0 μmol/L (IQR 38.7–127.0 μmol/L) in all subtypes (n = 67), 115.5 μmol/L (IQR 54.5–308.0 μmol/L) was in OTCD patients (n = 20) with the most significant deviation (P < 0.05). Among these UCD patients, 7 had blood ammonia levels exceeding 360.0 μmol/L, 5 had levels between 180.0 and 360.0 μmol/L, and the remaining patients had levels below 180.0 μmol/L.

**Table 4:** Biochemical results of different UCD subtypes at diagnosis

	Amon (μmol/L)	ALT (U/L)	AST (U/L)	Cit (μmol/L)	Arg (μmol/L)	Orn (μmol/L)	Gly (μmol/L)	Val (μmol/L)	Leu (μmol/L)	Hb (g/L)
OTCD (n = 35)	115.5 (54.5–308.0)	171.0 (25.5–390.9)	50.0 (30.3–195.8)	13.0 (4.3–20.2)	10.2 (6.9–17.2)	71.6 (57.1–130.4)	298.0 (203.6–443.2)	125.2 (110.8–139.7)	117.7 (91.7–171.1)	133.5 (116.8–147.5)
ASSD (n = 25)	42.0 (29.0–91.0)	19.5 (16.0–28.8)	40.0 (32.5–52.3)	197.2 (96.4–1253.3)	11.7 (6.7–25.2)	117.2 (88.8–154.1)	463.9 (376.7–615.0)	153.4 (128.4–175.6)	163.8 (147.2–198.3)	117.0 (105.0–136.3)
CPS1D (n = 13)	62.0 (49.6–132.0)	33.0 (25.0–47.0)	38.0 (30.0–52.7)	3.8 (3.3–6.2)	4.9 (3.0–12.9)	84.8 (70.8–90.5)	353.2 (285.4–437.5)	139.9 (110.9–162.4)	142.4 (132.1–202.5)	110.0 (91.0–126.8)
ARG1D (n = 12)	52.0 (36.5–59.3)	29.5 (16.8–37.3)	39.5 (33.0–70.5)	19.2 (16.0–26.6)	149.4 (84.8–309.2)	76.5 (44.1–105.5)	537.4 (411.2–644.9)	133.7 (115.9–159.8)	139.9 (130.8–178.3)	ND (ND-99.0)
ASLD (n = 10)	59.5 (39.8–109.3)	43.0 (28.5–78.5)	49.0 (29.0–56.0)	110.7 (96.4–148.1)	9.1 (7.3–14.9)	76.1 (48.4–125.3)	494.8 (346.1–576.7)	150.1 (143.0–157.1)	147.6 (144,8–151.1)	137.0 (128.0–147.0)
HHHS (n = 6)	64.0 (51.2–71.0)	467.7 (248.7–526.5)	100.6 (91.9–331.6)	27.3 (24.6–28.6)	11.4 (6.8–11.9)	199.6 (139.9–249.2)	236.4 (203.6–443.2)	161.7 (129.1–164.2)	137.2 (115.2–164.9)	161.0 (142.0–180.0)

Median alanine transferase (ALT) level was 171.0 U/L (IQR 25.5–390.9 U/L) in OTCD patients (n = 35), also found notably higher than other subtypes (P = 0.014). Median aspartate transferase (AST) levels were 100.6 U/L (IQR 91.3–331.6 U/L) and 50.0 U/L (IQR 30.3–195.8 U/L) in HHHS (n = 6) and OTCD (n = 35) patients, respectively, which were significantly elevated compared to other subtypes (P < 0.001, P < 0.001). The median citrulline (Cit) level was 197.2 μmol/L (IQR 96.4–1253.3 μmol/L) in ASSD (n = 25) and was significantly elevated compared to 110.7 μmol/L (IQR 96.4–148.1 μmol/L) in ASLD patients (n = 10) (P < 0.001). In contrast, OTCD and CPS1D patients had lower Cit levels, with medians of 13.0 μmol/L (IQR 4.3–20.2 μmol/L) and 3.8 μmol/L (IQR 3.3–6.2 μmol/L), respectively. Additionally, arginine (Arg) levels were significantly elevated in ARG1D patients (P < 0.001), with a median level of 149.4 μmol/L (IQR 84.8–309.2 μmol/L). On the other hand, ornithine (Orn), glycine (Gly), valine (Val), and leucine (Leu) levels were generally within the normal range across all subtypes, showing no significant deviations. Hemoglobin (Hb) levels, however, were slightly below the normal range in CPS1D, ARG1D, and ASLD patients, as shown in Table 4.

### Genetic mutation analysis

Only 65 patients provided genetic testing reports, and a total of 88 mutations with 59 (67.0%) mutations for missense, 12 (14.0%) for splice site, 9 (10.0%) for nonsense mutations (PTC), 7 (8.0%) for deletions, and 1 (1.0%) for duplication were found as detailed in Table 5.

**Table 5:** Detailed mutation information in UCD genes for 65 patients

Gene	Exon/Intron	Nucleotide change	Amino acid change	Type	Nature of mutation
*OTC*	Intron 1	c.77+5G>A	Splice site	Splice site	NA
	Intron 5	c.514-35C>G	Splice site	Splice site	Inherited
	Intron 6	c.663+1G>A	Splice site	Splice site	Inherited
	Intron 8	c.867+1G>A	Splice site	Splice site	Inherited
	Exon 1	c.67C>T	p.R23*	PTC	De novo
	Exon 2	c.115G>T	p.G39C	Missense	Inherited
	Exon 2	c.119G>A	p.R40H	Missense	NA
	Exon 2	c.140dupA	p.N47Kfs*8	PTC	NA
	Exon 4	c.353dup	ND	Duplication	NA
	Exon 5	c.392T>C	p.L131S	Missense	Inherited
	Exon 5	c.485G>A	p.G162E	Missense	Inherited
	Exon 6	c.586G>T	p.D196Y	Missense	De novo
	Exon 6	c.587A>T	p.D196V	Missense	De novo
	Exon 6	c.594C>G	p.N198K	Missense	Inherited
	Exon 6	c.602T>C	p.L201P	Missense	Inherited
	Exon 6	c.641A>G	p.H214R	Missense	Inherited
	Exon 8	c.799A>C	p.S267R	Missense	Inherited
	Exon 8	c.870delT	p.A291Lfs*32	PTC	Inherited
	Exon 9	c.912G>T	p.L304F	Missense	De novo
	Exon 9	c.929_931del	p.E310del	Deletion	Inherited
	Exon 9	c.931G>A	p.V311M	Missense	De novo
	Exon 10	c.1019C>T	p.S340F	Missense	Inherited
	Exon1-10	Exon1-10del	ND	Deletion	NA
*CPS1*	Intron 14	c.1549+1G>T	Splice site	Splice site	Inherited
	Exon 4	c.390_403del	p.L132*	PTC	Inherited
	Exon 8	c.862G>T	p.G288W	Missense	NA
	Exon 14	c.1436C>T	p.A479V	Missense	NA
	Exon 17	c.1864G>A	p.V622M	Missense	Inherited
	Exon 19	c.2228T>C	p.I743T	Missense	NA
	Exon 19	c.2287G>T	p.D763Y	Missense	NA
	Exon 19	c.2359C>T	p.R787*	PTC	NA
	Exon 20	c.2372C>T	p.A791V	Missense	NA
	Exon 20	c.2441G>A	p.R814Q	Missense	Inherited
	Exon 23	c.2876A>G	p.Y959C	Missense	NA
	Exon 25	c.3031G>A	p.V1011M	Missense	Inherited
	Exon 28	c.3237del	p.L1081Cfs*19	PTC	NA
	Exon 28	c.3443T>A	p.M1148K	Missense	NA
*ASS1*	Intron 3	c.174+1G>A	Splice site	Splice site	NA
	Intron 5	c.421-2A>G	Splice site	Splice site	Inherited
	Intron 10	c.773+4A>C	Splice site	Splice site	Inherited
	Intron 12	c.970+5G>A	Splice site	Splice site	Inherited
	Intron 12	c.970+5G>T	Splice site	Splice site	NA
	Exon 2	c.53C>T	p.S18L	Missense	NA
	Exon 3	c.133G>A	p.E45K	Missense	Inherited
	Exon 3	c.151G>A	p.A51T	Missense	Inherited
	Exon 3	c.173A>G	p.N58S	Missense	Inherited
	Exon 3	Exon 3del	ND	Deletion	Inherited
	Exon 4	c.256C>T	p.R86C	Missense	Inherited
	Exon 6	c.431C>G	p.P144R	Missense	NA
	Exon 7	c.544G>A	p.D182N	Missense	Inherited
	Exon 9	c.577G>A	p.G193R	Missense	Inherited
	Exon 9	c.694C>T	p.P232S	Missense	Inherited
	Exon 10	c.748C>T	p.L250F	Missense	Inherited
	Exon 10	c.772G>A	p.A258T	Missense	Inherited
	Exon 11	c.836G>A	p.R279Q	Missense	NA
	Exon 12	c.851C>T	p.T284I	Missense	Inherited
	Exon 12	c.919C>T	p.R307C	Missense	Inherited
	Exon 12	c.920G>A	p.R307H	Missense	Inherited
	Exon 13	c.1004G>A	p.R335H	Missense	Inherited
	Exon 13	c.1087C>T	p.R363W	Missense	Inherited
	Exon 14	c.1142G>A	p.G381D	Missense	Inherited
	Exon 14	c.1168G>A	p.G390R	Missense	Inherited
	Exon 14	c.1176_1178del	p.392_392de1	Deletion	Inherited
	Exon14-15	Exon14-15del	ND	Deletion	Inherited
*ASL*	Intron 10	c.719-1G>C	Splice site	Splice site	Inherited
	Exon 3	c.91G>A	p.D31N	Missense	Inherited
	Exon 4	c.269C>T	p.T90I	Missense	Inherited
	Exon 5	c.331C>T	p.R111W	Missense	Inherited
	Exon 6	c.430G>A	p.E144K	Missense	NA
	Exon 6	c.467C>T	p.P156L	Missense	Inherited
	Exon 6	c.502C>T	p.R168C	Missense	Inherited
	Exon 7	c.503G>A	p.R168H	Missense	Inherited
	Exon 8	c.544C>T	p.R182*	PTC	Inherited
	Exon 10	c.706C>T	p.R236W	Missense	Inherited
	Exon 11	c.772G>A	p.E258K	Missense	Inherited
*ARG1*	Intron 5	c.560+2T>C	Splice site	Splice site	De novo
	Exon 2	c.130G>T	p.E44*	PTC	Inherited
	Exon 3	c.212G>C	p.R71T	Missense	Inherited
	Exon 4	c.383A>T	p.D128V	Missense	Inherited
	Exon 5	c.466-5delTT	ND	Deletion	Inherited
	Exon 5	c.466-6_466-5del	ND	Deletion	Inherited
	Exon 7	c.703G>A	p.G235R	Missense	De novo
	Exon 7	c.775T>C	p.Y259H	Missense	Inherited
	Exon 8	c.922C>T	p.R308W	Missense	Inherited
*SLC25A15*	Exon 3	c.255C>G	p.Y85X	Missense	NA
	Exon 3	c.268C>T	p.Q90*	PTC	Inherited
	Exon 5	c.535C>T	p.R175X	Missense	NA
	Exon 7	c.815C>T	p.T272I	Missense	Inherited

In the 23 OTCD patients, 23 hemizygous mutations were identified. While most mutations in this gene were inherited, a few de novo mutations, such as c.586G > T (p.D196Y), were also observed. The findings indicated that patients with OTCD primarily exhibited gastrointestinal symptoms. In the *CPS1* gene, c.1864G > A (p.V622M) and c.3031G > A (p.V1011M) were the two homozygous mutations were identified, along with two heterozygous and five compound heterozygous mutations. Apart from three PTCs and one splice site mutation, the remaining 10 mutations were missense mutations. In the *ASS1* gene, four mutations were recurrent, with c.1168G > A (p.G390R) appearing in three patients, including the only homozygous mutation observed among ASSD patients. Additionally, there were three heterozygous mutations and 14 compound heterozygous mutations, with missense mutations being the most common, totaling 19 cases. The *ASL* gene showed two recurrent mutations, each appearing twice. Aside from one homozygous mutation, c.331C > T (p.R111W), all others were compound heterozygous. The mutations included one splice site mutation, one PTC, and the remainder were missense mutations. In the *ARG1* gene, the c.703G > A (p.G235R) mutation was identified in two cases. No homozygous mutations were observed, with all cases being compound heterozygous. Similarly, the *SLC25A15* gene had no homozygous mutations, presenting with two heterozygous and two compound heterozygous mutations. While those with CPS1D, ASSD, ASLD, and HHHS were more commonly associated with neurological symptoms or abnormal laboratory results.

Of the total number of variants detected, 17(17/88, 19.0%) novel mutations were identified in 14 patients. These novel mutations were defined as variants not previously reported in public databases such as ClinVar, gnomAD, or HGMD, nor in the published literature. We employed prediction tools to assess the potential pathogenicity of these novel mutations (Table 6). These mutations exhibited varying degrees of predicted pathogenicity. For instance, the missense mutations c.694C > T (p.P232S) in the *ASS1* gene and c.383A > T (p.D128V) in the *ASL* gene were classified as damaging or potentially damaging by multiple prediction tools. Conversely, some mutations, such as c.173A > G (p.K58R) in the *ASS1* gene, were predicted to be benign or tolerated.

**Table 6:** Predicted pathogenicity of novel mutations in UCD genes

Gene	Nucleotide change	Amino acid change	SIFT^a^	PROVEAN^b^	PolyPhen-2^c^	Mutation taster^d^
*OTC*	c.353dup	p.L118Ffs*5	Damaging	Deleterious	NA	NA
*CPS1*	c.862G>T/c.2287G>T	p.E288*/p.E763*	NA/NA	Neutral/Deleterious	NA/NA	6/6
*ASS1*	Exon14-15del	NA	NA	NA	NA	NA
*ASS1*	c.694C>T	p.P232S	Damaging	Deleterious	Possibly damaging	74
*ASS1*	Exon3del	NA	NA	NA	NA	NA
*ASS1*	c.1142G>A	p.G381D	Tolerated	Deleterious	Possibly damaging	94
*ASS1*	c.173A>G	p.K58R	Tolerated	Neutral	Benign	26
*ASL*	c.430G>A	p.V144M	Damaging	Neutral	Benign	21
*ASL*	c.269C>T	p.T90I	Damaging	Deleterious	Possibly damaging	89
*ARG1*	c.466-6_466-5del	NA	NA	NA	NA	NA
*ARG1*	c.130G>T/c.383A>T	p.E44*/p.D128V	NA/Damaging	Deleterious/Deleterious	NA/Possibly damaging	6/152
*ARG1*	c.775T>C/c.466-5delTT	p.Y259H/splicing	Damaging/NA	Deleterious/NA	Benign/NA	83/NA
*SLC25A15*	c.255C>G	p.Y85X	NA	Deleterious	NA	6
*SLC25A15*	c.268C>T	p.Q90*	NA	Deleterious	NA	6

^a^SIFT prediction: Amino acids with probabilities < 0.05 are predicted to be deleterious, whereas variants with a score above 0.05 are considered"tolerated"

^b^PROVEAN prediction: Variants with scores below − 2.5 are considered deleterious, while variants with scores above − 2.5 are considered neutral

^c^PolyPhen-2 prediction: Variants with scores close to 1 are considered"probably damaging", while those with scores below 1 may be"possibly damaging"or"benign"

^d^MutationTaster prediction: Scores range from 0.0 to 215. Higher scores indicate more deleterious effects

### Long-term clinical outcomes

We conducted a statistical analysis of the clinical outcomes in UCD patients, focusing on survival rates, liver transplantation, intellectual and motor development.

The survival rate was 93.0% (94/101); among the deceased patients, 3 had OTCD, 2 had ASSD, 1 had CPS1D, and 1 had ARG1D. A total of 7 patients (7/101, 7.0%) had undergone liver transplantation (5 patients with OTCD and 2 for CPS1D). 19 patients (19/101, 19.0%) had suffered intellectual and motor impairments, including 7 cases of OTCD, followed by CPS1D and ASLD in 4 cases each, 2 cases of HHHS, and ASSD and ARG1D in 1 case each.

Additionally, we conducted a subgroup analysis comparing clinical outcomes and nitrogen scavenger usage between LO group and NO group (Table 3). The survival rates were 86.0% (30/35) and 98.0% (57/58) in LO and NO group, respectively, and there was a statistically significant difference between the two groups (P = 0.032). 11 patients (11/20, 55.0%) and 8 patients (8/44, 18.0%) had suffered the intellectual and motor impairments even after long-term management in LO and NO group, respectively, the incidence of that impairments was also significantly higher in the LO group compared to the NO group (P = 0.003), with the analysis excluding patients with unknown outcomes. However, there were no significant differences between the two groups regarding liver transplantation. Notably, 13 patients (13/23, 56.0%) had reported to use nitrogen scavengers in LO group, while only 8 patients (8/37, 22.0%) in NO group, which made a significantly difference (P = 0.006). This analysis also excluded patients with unknown outcomes.

### Biochemical analysis results during follow-up

**Fig. 4:**
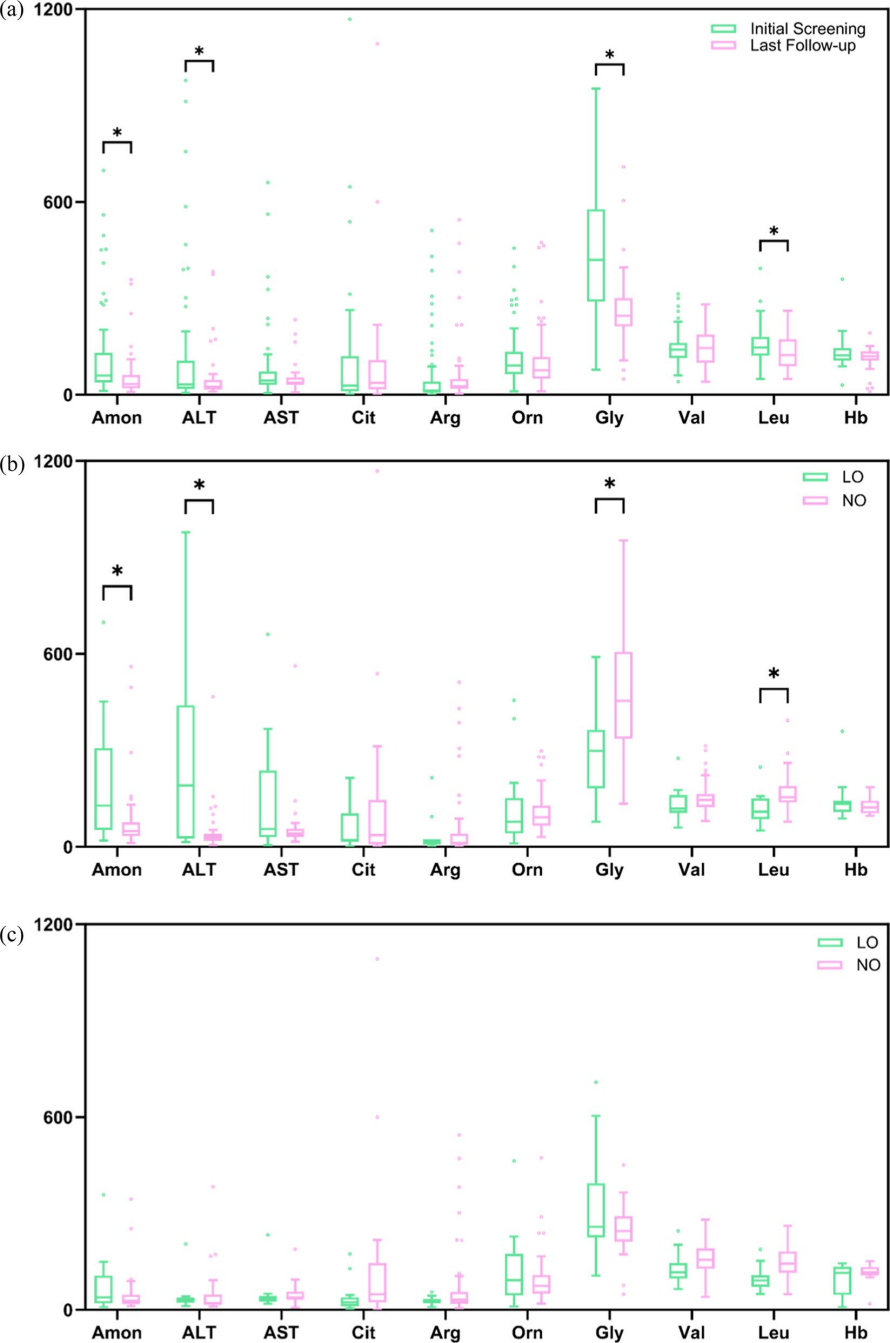
Biochemical results in UCD patients. **a** Initial screening vs. last follow-up biochemical markers. **b** LO vs. NO groups in baseline. **c** LO vs. NO groups in last visit

Figure 4a showed the comparisons biochemical results in UCD patients (including all patients, regardless of whether they received drug therapy or dietary therapy) between the initial screening and the last visit after treatment. Compared to the initial screening, blood ammonia levels significantly decreased at the last visit (P < 0.01), with the median dropping from 60.0 μmol/L (IQR 38.8–129.0 μmol/L) to 33.5 μmol/L (IQR 21.2–56.0 μmol/L). At last visit, the ALT levels significantly reduced from a median of 32.0 U/L (IQR 18.3–98.3 U/L) to 25.0 U/L (IQR 16.5–45.0 U/L) (P = 0.021), and although the values of AST were also falling, no significant difference were observed compared to baseline. As for branched-chain amino acid (BCAA), only Leu levels were found to decrease significantly (P = 0.036), with the median level of 147.5 μmol/L (IQR 122.5–179.3 μmol/L) at baseline and 123.8 μmol/L (IQR 91.1–171.4 μmol/L) at last visit, while the median of Val values rose from 140.7 μmol/L (IQR 115.8–161.7 μmol/L) to 145.9 μmol/L (IQR 101.9–184.0 μmol/L). Gly levels were substantial reduce (P < 0.001) from the median level of 419.7 μmol/L (IQR 293.5–571.8 μmol/L) to 246.2 μmol/L (IQR 215.6–298.8 μmol/L). Both Cit and Arg levels increased modestly and showed no significant trend between the baseline and the last visit, the median value of Cit and Arg were 28.6 μmol/L (IQR 10.9–116.1 μmol/L) and 13.3 μmol/L (IQR 6.6–41.2 μmol/L) at the baseline, 37.1 μmol/L (IQR 18.4–107.2 μmol/L) and 26.6 μmol/L (IQR 18.9–48.4 μmol/L)at the last visit, respectively.

The further analyzed about biochemical results between the LO group and NO group in terms of baseline were shown in Fig. 4b. The median blood ammonia level in the LO group was 129.0 μmol/L (IQR 52.9–294.0 μmol/L), and 49.0 μmol/L (IQR 33.8–73.7 μmol/L) in NO group, which was significantly lower than LO group (P = 0.008). Liver function markers were generally elevated in the LO group compared to the NO group, with ALT levels. The median ALT level was 67.0 U/L (IQR 18.8–368.0 U/L) in LO group, compared to 29.5 U/L (IQR 19.0–39.0 U/L) in the NO group, showing the significant difference (P = 0.016). It was found that the median Gly level in the LO group was significantly lower than the NO group (P = 0.014), which were 346.4 μmol/L (IQR 232.0–470.3 μmol/L) and 454.8 μmol/L (IQR 339.3–589.3 μmol/L), respectively. And the median Leu levels were also significantly lower in the LO group (P = 0.001), as 106.9 μmol/L (IQR 88.1–144.4 μmol/L) compared to 155.0 μmol/L (IQR 138.6–188.6 μmol/L) in the NO group. However, there were no statistically significant differences between the LO and NO groups about the biochemical results at the last visit (Fig. 4c).

### Growth and neurological outcomes

**Fig. 5:**
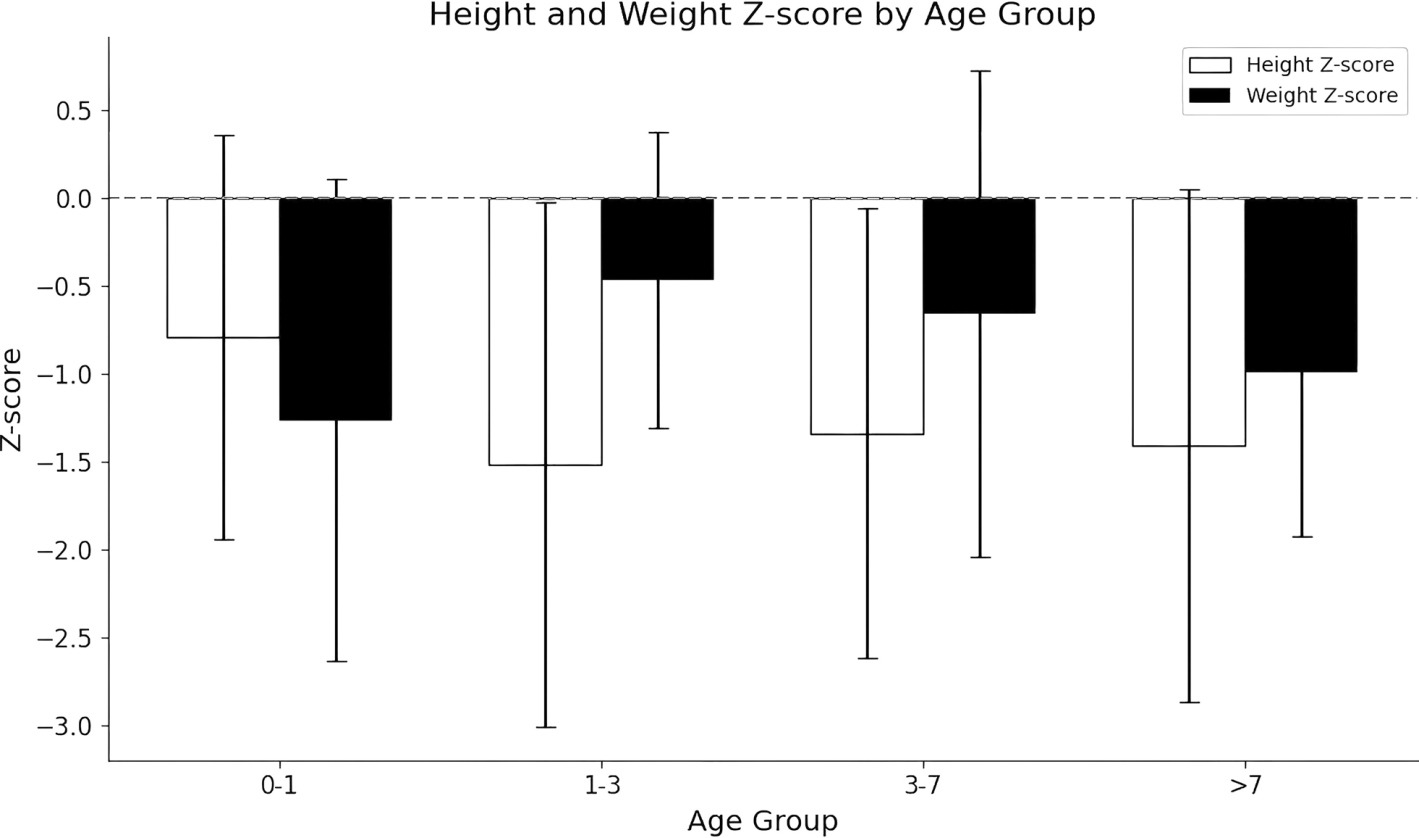
Height and weight Z-scores by age group for UCDs patients. Normal (− 2 to + 2): Height or weight is within the typical range. Low (< − 2): Indicates potential growth delays or malnutrition. High (> + 2): Indicates potential rapid growth or overweight

Delayed physical growth affected 49.0% of all patients, particularly notable in 66.0% (23/35) of OTCD, 69.0% (9/13) of CPS1D and 83.0% (5/6) of HHHS patients. Height and weight average Z-score in all age groups were within the normal range (− 2 to + 2) (Fig. 5). However, both Z-score below the average levels for children with corresponding age, particularly the weight Z-score in < 1 year age group and the height Z-score in > 1 year age group. Intellectual and motor impairments were not assessed using psychometric scales, but rather based on neuroimaging. Only 34 UCD patients underwent brain magnetic resonance imaging, and the results revealed that 28 patients exhibited significant neurological symptoms, such as seizures. Similarly, a subgroup analysis was conducted to compare growth retardation and neurological abnormalities between the LO and NO groups. There were no significant difference in growth retardation, but neurological abnormalities were more severe in the LO group than in the NO group (P = 0.001).

## Discussion

Our study was the first in China to describe the characteristics and long-term outcomes of UCD patients. The majority of patients in our study were from eastern and southern China, which is likely related to the greater application of tandem mass spectrometry in the southeastern region, in addition to our study center being located in Zhejiang province [1]. Notably, 57.0% of the cases in this study were found through NBS, with Zhejiang province accounting for 31.0% of these cases (18/58). Due to NBS, 30 out of 101 patients (29.0%) in our study were diagnosed within the first month of life, highlighting the impact of early screening on timely diagnosis. OTCD was always the most common subtype across all six UCD variants, accounting for 34.0% of cases with an almost equal gender distribution [21]. It’s widely known that the detection rate to proximal UCDs by NBS was relatively low, particularly in OTCD, which may be attributed to non-specific biochemical markers, X-linked compensation effects, and delayed symptom onset [22]. However, our data indicated that 7 CPS1D (7/13, 53.8%) and 9 OTCD (9/35, 25.7%) patients were detected via NBS. These results emphasize the importance of widespread newborn screening programs for early UCD detection, including proximal subtypes. Additionally, high-throughput sequencing and multi-omics strategies are increasingly recognized for enhancing screening sensitivity and detecting complex cases [23–26].

Among the seven patients who appeared symptoms before NBS, four of which died in the neonatal period due to severe hyperammonemia, suggesting that early diagnosis may limited in cases onset very early [21]. Additionally, approximately 50.0% of infants UCD patients experience hyperammonemia, with a mortality rate ranging from 25.0 to 50.0% [10]. But our data shown the mortality rate of infants UCD patients was 57.1%, slightly higher in our cohort. This highlights the urgently need to improve the diagnostic, treatment, and management capabilities for UCDs in China. Moreover, neurological sequelae are more common in early-onset patients, who may also experience recurrent hyperammonemia [5]. Among the seven early-onset patients, one case presented with severe hyperammonemia, including coma, seizures. However, after aggressive treatment, the prognosis has been very favorable. This highlights that while early diagnosis is challenging, timely identification and urgent intervention for unexplained hyperammonemia are crucial to reduce mortality in severe cases.

Excepted for ammonia levels, specific biochemical pattern in some UCD subtypes are crucial for early diagnosis, facilitating faster identification and management of patients [10]. For instance, the hallmark biochemical feature of ARG1D patients is a significant elevation in plasma Arg levels [27]. In our study, plasma Arg levels in ARG1D patients were 149.4 μmol/L (IQR 84.8–309.2 μmol/L), significantly higher than in other subtypes. Cit is a key marker in ASSD, while argininosuccinic acid (ASA) is the primary marker in ASLD. Both disorders are associated with elevated Cit levels, but ASSD typically shows levels above 300.0 μmol/L, while ASLD levels are generally above 100.0 μmol/L. This difference may be related to the stage at which disruptions occur in the urea cycle [28, 29]. In our study, the Cit levels in ASSD and ASLD patients were 197.2 μmol/L (IQR 96.4–1253.3 μmol/L) and 110.7 μmol/L (IQR 96.4–148.1 μmol/L). It is important to note that in ASLD patients, the increase in Cit is relatively small, and in some cases, it may appear normal in NBS, leading to a significant number of missed diagnoses. In contrast, Cit levels in NBS are much higher in ASSD patients. Therefore, we recommend Cit as a distinguishing marker for both subtypes. However, in our patient cohort, there were some ASSD patients with abnormally low Cit levels and some ASLD patients with exceptionally high Cit levels. Hence, we recommend incorporating ASA as a key method to differentiate between these two subtypes in NBS indicators, as ASA is absent in ASSD but elevated in ASLD [10]. In our study, Cit levels in CPS1D and OTCD patients were lower than in other subtypes, but the difference was minimal. Accurate differentiation requires urine organic acid tests and genetic analysis. In HHHS patients, Orn levels were within the normal range but higher than in other subtypes, serving as a potential diagnostic indicator.

We conducted a screening for hotspot mutations across different UCD subtypes but did not identify the commonly observed hotspot mutations in genes like *OTC*. The distribution of gene mutations among UCD patients was highly scattered, which might be related to genetic differences across research populations and regional genetic backgrounds. For example, the *OTC* gene mutation c.119G > A (p.R40H) was more commonly found in studies from Japan [30]. Meanwhile, c.1168G > A (p.G390R) was a global hotspot mutation in the *ASS1* gene, reported in studies from multiple countries [31]. Although our study did not find these commonly reported hotspot mutations, the results highlight the high genetic heterogeneity of UCD gene mutations across different populations. Studies have shown that mutant proteins can disrupt the formation, function, or stability of protein complexes through dominant-negative effects or interfere with the interaction of wild-type proteins, thereby impairing their normal function. For example, mutations such as p.G390R and p.R363W have been found to affect the oligomerization of the ASS1 enzyme [32]. Based on three patients with p.G390R mutations in our cohort, we found that this mutation is associated with frequent hyperammonemia crises, highlighting its importance in management. Additionally, although UCD is mostly inherited in an autosomal recessive pattern, a compound heterozygous mutation is not always required for diagnosis. In our cohort, except for OTCD (X-linked inheritance) and ARG1D patients (both of whom had compound heterozygous mutations), other subtypes had patients with a single mutation. Even with one mutation and no obvious symptoms, a diagnosis could be made based on biochemical features like elevated ammonia levels and amino acid abnormalities. For patients without genetic testing, an initial diagnosis could still be made using clinical and biochemical data for timely intervention.

Due to individual differences, the treatment of UCD needs to be tailored according to the patient's condition. Low-protein diet combined with citrulline and arginine supplementation is a conventional treatment approach. Nitrogen scavengers, especially GPB, are effective in controlling hyperammonemia, but their use in China is still limited by cost and availability [33–36]. Subgroup analysis showed that more LO patients used nitrogen scavengers than NO patients (P < 0.05), indicating more severe conditions requiring pharmacological intervention. In addition to nutritional and pharmacological treatments, there are patients who undergo liver transplantation. In our study, 7 patients who underwent liver transplantation were all with proximal UCDs (5 for OTCD, 2 for CPS1D), which aligns with our findings of more severe metabolic disruptions in these subtypes. The average age at transplantation was 2 years and 5 months, with the oldest patient being 6 years and 9 months. According to Chinese guidelines, early-onset UCD patients are recommended to undergo liver transplantation as early as possible, preferably before the age of one, when their condition is stable [37]. Although liver transplantation plays a crucial role in the treatment of UCD, the decision should be based on the patient’s clinical condition and metabolic control. Even in some severe early-onset cases, if metabolic control is well-maintained, liver transplantation may not be necessary. Consequently, our liver transplantation rate was relatively low, at 6.9% (7/101). In contrast, liver transplantation rates are higher in other countries. For example, a retrospective study in Japan reported a transplantation rate of 33.8% (78/231) among UCD patients [38]. This suggests that the relatively low rate of liver transplantation in our cohort may be associated with early management strategies, including timely diagnosis and metabolic control. Post-transplant, ammonia levels in all seven patients were well-controlled with no further hyperammonemia episodes, and their metabolic status stabilized. Some patients showed improvements in neurological symptoms, such as seizures and lethargy. However, three patients with pre-transplant neurological impairment did not recover cognitive function. This indicates that while liver transplantation cannot fully reverse pre-existing neurological damage [5, 38], it can still contribute to partial neurological improvement and enhance quality of life.

Long-term biochemical data showed that available treatments effectively control blood ammonia levels and address metabolic issues. In 101 UCD patients from China, the average annual hospitalization rate due to hyperammonemia was 0.91 times per patient, with a peak blood ammonia level of 697.8 ± 475.1 µmol/L. The median blood ammonia level in the LO group was significantly higher than the NO group (P = 0.008), but there were no significant differences post-treatment, indicating that timely intervention improves outcomes. Our study found a significant decrease in Leu levels after treatment, likely related to nutritional and drug management, as many patients had excessive restriction of protein intake, and 9.0% (6/67) of patients were treated with NaPBA, which is known to reduce BCAA levels [39], and also associated with the lack of timely monitoring and supplementation of BCAA during long-term management. Therefore, it is recommended that patients with low BCAA levels should consume high-quality protein, monitor BCAA levels regularly, and supplement BCAAs promptly to make sure the children growth and development [40]. Similarly, the significant decrease in Gly levels after treatment may be associated with the excessively low-protein diet, or the usage of NaBz treatment, which works by binding with glycine to form a glycine conjugate, thereby facilitating the reduction of blood ammonia levels [41]. If the blood Gly falls below 100.0 µmol/L, the dosage of NaBz should be reduced, or an alternative nitrogen scavenger should be considered [37]. Furthermore, current studies have not identified a significant impact of GPB on BCAA levels [42, 43].

In our study, intellectual and motor developmental impairments were observed in a significant proportion of UCD patients (19.0%), and 28.0% of patients exhibited neuroimaging abnormalities. These impairments had real-life consequences, as reflected by the fact that some children required enrollment in special education institutions. This indicates that neurotoxic effects of hyperammonemia would result in long-term detrimental effects on the nervous system of UCD patients [44]. Further analysis showed that neurological damage was significantly more severe in the LO group compared to the NO group (P = 0.003), which may be associated with higher blood ammonia levels observed at the first episode in the LO group, and previous studies also have suggested that patients with higher blood ammonia levels (≥ 360.0 μmol/L) during their first episode of the disease may experience poorer neurological recovery [7]. So early diagnosis and rapid blood ammonia reduction play a crucial role in protecting the nervous system and improving outcomes. However, Intellectual and motor impairments were assessed based on neuroimaging findings and clinical symptoms, rather than using psychometric scales, which is a limitation of this study.

As a result, the limitations of this study include: (1) It is retrospective design; (2) The sample size is relatively small, with some subtypes having limited sample representation; (3) Nutritional management (protein and calorie intake) was not collected or included in the analysis; (4) Neurocognitive outcomes were assessed qualitatively rather than quantitatively. Nonetheless, the study provides valuable insights into the clinical spectrum, management strategies, and outcomes of UCD patients in China.

## Conclusion

This is the first study to systematically describe the clinical features and long-term outcomes of UCD patients in China. A total of 7 deaths were reported, contributing to a 93.0% overall survival rate. 57.0% of cases were found through NBS. Neurological and gastrointestinal symptoms were common clinical symptom, elevated blood ammonia levels with blood amino acid profiling were help to diagnosed and distinguished UCD patients. 17 novel mutations were identified, expanding the genetic spectrum of UCDs. After a median follow-up of 3.7 years, all 7 patients who underwent liver transplantation were proximal UCDs. A low-protein diet combined with nitrogen scavengers effectively maintains stable blood ammonia and biochemical results. 49.0% of survivors experienced growth retardation. Late-onset patients had worse neurological impairments and a greater demand for nitrogen scavengers. All our findings highlight the necessity to promote newborn screening and enhance long-term management to improve prognosis for UCD patients in China.

## Supplementary Information

Additional file 1.

## Data Availability

The datasets used and/or analysed during the current study are available from the corresponding author on reasonable request.

## Abbreviations

UCDs: Urea cycle disorders
OTCD: Ornithine transcarbamylase deficiency
NAGSD: N-acetylglutamate synthase deficiency
CPS1D: Carbamoylphosphate synthetase 1 deficiency
ASSD: Argininosuccinate synthetase deficiency
ASLD: Argininosuccinate lyase deficiency
ARG1D: Arginase 1 deficiency
HHHS: Hyperornithinemia-hyperammonemia-homocitrullinuria syndrome
Citrin D: Citrin deficiency
NBS: Newborn screening
NaBz: Sodium benzoate
GPB: Glycerol phenylbutyrate
NaPBA: Sodium phenylbutyrate
LT: Liver transplantation
HA: Hyperammonemia
IQR: Interquartile range
ALT: Alanine transferase
AST: Aspartate transferase
Cit: Citrulline
Arg: Arginine
Orn: Ornithine
Gly: Glycine
Val: Valine
Leu: Leucine
Hb: Hemoglobin
BCAA: Branched-chain amino acid
ASA: Argininosuccinic acid
