# Supplementary material for: Clinical characteristics and long-term outcomes of 101 patients with urea cycle disorders in China
Source: Orphanet J Rare Dis. 2025 Aug 13;20:432. doi: 10.1186/s13023-025-03985-w (PMC12351844; doi:10.1186/s13023-025-03985-w)
Supplement: Supplementary file 1 — Additional file 1. [file 13023_2025_3985_MOESM1_ESM.docx]

| **Age at diagnosis** | **≤ 1 months** | **1 months to 1 year** | **1 year to 2 years** | **> 2 years** |
| --- | --- | --- | --- | --- |
| **OTCD** | 8 | 5 | 4 | 13 |
| **ASSD** | 11 | 6 | 2 | 1 |
| **CPS1D** | 6 | 4 | 2 | 0 |
| **ARG1D** | 4 | 4 | 1 | 0 |
| **ASLD** | 1 | 4 | 0 | 2 |
| **HHHS** | 0 | 2 | 0 | 2 |
| **Follow-up duration** | **< 1 year** | **1 to 2 years** | **2 to 5 years** | **> 5 years** |
| **OTCD** | 12 | 2 | 10 | 3 |
| **ASSD** | 6 | 7 | 2 | 4 |
| **CPS1D** | 3 | 3 | 0 | 2 |
| **ARG1D** | 4 | 1 | 2 | 2 |
| **ASLD** | 1 | 1 | 2 | 3 |
| **HHHS** | 1 | 0 | 2 | 0 |

**Supplementary Table 1** Age at Diagnosis and Follow-up Duration Stratified by UCD Subtypes.

This table summarizes the distribution of age at diagnosis and follow-up duration for each UCD subtypes. Note that some data are missing for certain patients, which may result in slight discrepancies in the totals.
